# A convolutional neural network based tool for predicting protein AMPylation sites from binary profile representation

**DOI:** 10.1038/s41598-022-15403-3

**Published:** 2022-07-06

**Authors:** Sayed Mehedi Azim, Alok Sharma, Iman Noshadi, Swakkhar Shatabda, Iman Dehzangi

**Affiliations:** 1 Department of Computer Science and Engineering, United International University, Plot-2, United City, Madani Avenue, Badda, Dhaka, 1212 Bangladesh; 2 Institute for Integrated and Intelligent Systems, Griffith University, Brisbane, Australia; 3 Laboratory for Medical Science Mathematics, RIKEN Center for Integrative Medical Sciences, Yokohama, 230-0045 Japan; 4 Department of Bioengineering, University of California, Riverside, CA 92507 USA; 5 Department of Computer Science, Rutgers University, Camden, NJ 08102 USA; 6 Center for Computational and Integrative Biology, Rutgers University, Camden, NJ 08102 USA

**Keywords:** Bioinformatics, Software, Computational biology and bioinformatics

## Abstract

AMPylation is an emerging post-translational modification that occurs on the hydroxyl group of threonine, serine, or tyrosine via a phosphodiester bond. AMPylators catalyze this process as covalent attachment of adenosine monophosphate to the amino acid side chain of a peptide. Recent studies have shown that this post-translational modification is directly responsible for the regulation of neurodevelopment and neurodegeneration and is also involved in many physiological processes. Despite the importance of this post-translational modification, there is no peptide sequence dataset available for conducting computation analysis. Therefore, so far, no computational approach has been proposed for predicting AMPylation. In this study, we introduce a new dataset of this distinct post-translational modification and develop a new machine learning tool using a deep convolutional neural network called DeepAmp to predict AMPylation sites in proteins. DeepAmp achieves 77.7%, 79.1%, 76.8%, 0.55, and 0.85 in terms of Accuracy, Sensitivity, Specificity, Matthews Correlation Coefficient, and Area Under Curve for AMPylation site prediction task, respectively. As the first machine learning model, DeepAmp demonstrate promising results which highlight its potential to solve this problem. Our presented dataset and DeepAmp as a standalone predictor are publicly available at https://github.com/MehediAzim/DeepAmp.

## Introduction

Post Translational Modification (PTM) is the enzymic or chemical modification of a protein after it is translated or synthesized in the ribosome. The PTMs are occurred via removal of parts of a translated protein, covalent modifications, or degradation of modified proteins^1,2^. These modifications provide important insight into various cellular functions and biological processes of proteins such as cellular dynamics and elasticity.

PTMs are important mechanisms to increase proteomic diversity, and play a vital role in functional proteomic because they regulate activity, localization, and interaction with other cellular molecules such as proteins, nucleic acids, lipids, and cofactors^3^. They can impact the structure, electrophilicity, and interactions of proteins. PTMs also regulate protein folding via targeting specific subcellular compartments, interacting with ligands or other proteins, or by initiating a change in their functional state including signaling or catalytic activity^4^. A wide range of PTMs have been identified so far. The common PTMs include phosphorylation, glycosylation, ubiquitination, nitrosylation, methylation, acetylation, lipidation, and proteolysis which influence almost all aspects of normal cell biology and pathogenesis^5^.

AMPylation is an emerging Post Translational Modification mediated by a bacterial virulence factor that transfers Adenosine Monophosphate (AMP) from Adenosine Triphosphate (ATP) to a threonine residue of eukaryotic substrates^6,7^. AMPylation is the covalent attachment of AMP to a protein or peptide^8^. It has been studied exclusively with the Fic domain proteins, which are preserved and found in proteins stretching from bacteria to humans. By adding AMP to Rho-family GTPases, these enzymes can thereby mediate both bacterial pathogenesis and eukaryotic signaling^9,10^. The most common and stable form of AMPylation occurs on the hydroxyl group of threonine, serine, or tyrosine via a phosphodiester bond. In the AMPylation process, Adenosine Monophosphate (AMP) gets covalently attached to the amino acid side chain of a protein molecule. AMPylation involves a phosphodiester bond between a hydroxyl group of the molecule undergoing AMPylation and the phosphate group of the adenosine monophosphate nucleotide (i.e. adenylic acid)^14^. The enzymes that are capable of catalyzing this process are called AMPylators. Threonine (T) and Tyrosine (Y) amino acids are usual targets of AMPylation while this PTM can sometimes be observed in Serine (S) as well.

Recent proteomics studies demonstrated that this PTM is more common than generally acknowledged and it is emerging as a significant regulatory mechanism for both eukaryotic and prokaryotic cells. It is impelled in a vast area of biological processes stretching from regulation of nitrogen metabolism in bacteria and regulation of signaling pathways to pathogenesis in several animal species^11–14^. AMPylation has also found to play a significant role in the regulation of neurodevelopment and neurodegeneration^15^.

Experimental approaches used to determine PTM sites are expensive, laborious, and time taking. Hence, many studies have been proposed to predict PTM sites using fast and cost effective computational approaches^16–28^. In 2015, Khater and Mohanty, used SVM and HMM to develop a computational protocol for identification of AMPylation domains and their classification into various functional subfamilies such as, catalyzing AMPylation, deAMPylation, phosphorylation, and phosphocholine transfer^29^. However, they did not directly predict if a given peptide is AMPylation or non-AMPylation.

To the best of our knowledge, so far no computational approach has been proposed for predicting AMPylation sites of Fic domain protein. One of the main reasons is that there is no AMPylation dataset available to be used for this task. In this study, we are presenting a new dataset of protein AMPylation sites. Furthermore, we also propose a new deep convolutional neural network (CNN) model called DeepAmp for predicting protein AMPylation sites on the newly found dataset of AMP modified proteins. DeepAmp achieves 77.7%, 79.1%, 76.8%, 0.55, and 0.85 in terms of Accuracy, Sensitivity, Specificity, Matthews Correlation Coefficient (MCC), and Area Under Curve (AUC) for AMPylation site prediction task, respectively. As the first machine learning model, DeepAmp demonstrate promising results which highlight its potential to solve this problem. We believe this study will help researchers immensely in terms of mitigating the current research gap in this subject. Our presented dataset and DeepAmp as an standalone predictor are publicly available at https://github.com/MehediAzim/DeepAmp.

## Results and discussion

### Evaluation metrics

In order to ensure standardized evaluation of our model and to provide more insights into our results, we calculate the Accuracy, Sensitivity, Specificity, and Mathews correlation coefficient (MCC) as the evaluation metrics. These metrics are characterized by the following equations:

1 $$\begin{aligned} Accuracy= & {} \frac{tp+tn}{tp+tn+fp+fn} \times 100, \end{aligned}$$

2 $$\begin{aligned} Sensitivity= & {} \frac{tp}{tp + fn} \times 100, \end{aligned}$$

3 $$\begin{aligned} Specificity= & {} \frac{tn}{tn + fp} \times 100, \end{aligned}$$

4 $$\begin{aligned} MCC= & {} \frac{(tp \times tn) - (fp \times fn)}{\sqrt{(tp + fp)(tp + fn)(tn + fp)(tn + fn)}}, \end{aligned}$$

where tp denotes true positive, and tn, fp, fn denote true negative, false positive, and false negative, respectively.

Additionally, to show the model’s distinguishing capability between AMPylated and non-AMPylated sites, we calculated the Area Under the Curve (AUC). AUC measures the ability of a classifier to distinguish between classes. The higher the AUC, the better the performance of the model at distinguishing between the positive and negative instances. AUC value of 1 indicates that the classifier can differentiate between all the Positive and the Negative class points, correctly. While, an AUC value of 0 indicates poor performance^30^.

### Comparison with different machine learning techniques

Since DeepAmp is the first computational model proposed to predict AMPylation PTM, it is not possible to compare model performance with any other studies. However, to investigate the effectiveness of CNN to build DeepAmp, we compare it with other ML models to solve this problem. Results achieved using DeepAmp compared to other ML models including Support Vector Machine (SVM), Random Forest (RF), Linear Regression (LR), Decision Tree (DT), and K-Nearest Neighbor (KNN) using same set of features are presented in Tables 1 and 2 for fivefold and tenfold cross-validations, respectively. We present the average of 10 runs of fivefold and tenfold cross-validations model for all the metrics in Tables 1 and 2. As shown in these tables, DeepAmp achieves significantly better results in terms of all four metrics than other machine learning methods which are investigated in this study.

As shown in Table 1, DeepAmp achieves 75.9%, 77.2%, 75.2%, 0.52, and 0.84 in terms of Accuracy, Sensitivity, Specificity, MCC, and AUC for AMPylation site prediction task using fivefold cross validation, respectively. Also, according to Table 2, DeepAmp achieves 77.7%, 79.1%, 76.8%, 0.55, and 0.85 in terms of Accuracy, Sensitivity, Specificity, MCC, and AUC for AMPylation site prediction task using tenfold cross validation, respectively. As shown in these Tables, the prediction accuracy and MCC of other classifiers explored in this study are all below 70.0% and 0.30, respectively which demonstrate the effectiveness of using CNN to build DeepAmp.

**Table 1:** Different models result on AMPylation (fivefold CV).

Model	Accuracy	Sensitivity	Specificity	MCC	AUC
DeepAMP	75.9	77.2	75.2	0.52	0.84
LR	65.5	49.0	75.6	0.26	0.68
SVM	62.5	47.7	71.6	0.20	0.65
DT	59.6	51.0	64.8	0.16	0.57
KNN	46.4	86.3	22.0	0.10	0.55
RF	66.0	39.9	82.0	0.25	0.67

As shown in Tables 1 and 2, the results using tenfold cross-validation are slightly better than those reported using fivefold cross-validation. This can be associated with larger number of samples used to train our model in tenfold cross-validation. In k-fold we evaluate the model on 1/k part of the data and train on the rest. Therefore, for our dataset, in tenfold we are using 362 samples for training while in fivefold we are using 320 samples for training in each iteration. As a result, there are more samples available to train the model using tenfold rather than 5-fold. This also suggest that by having a larger dataset, DeepAmp is able to achieve even better results.

**Table 2:** Different models result on AMPylation dataset (10 fold CV).

Model	Accuracy	Sensitivity	Specificity	MCC	AUC
DeepAMP	77.7	79.0	76.8	0.55	0.85
LR	66.2	51.0	75.6	0.28	0.69
SVM	65.0	51.6	73.2	0.26	0.66
DT	61.7	61.4	62.0	0.23	0.67
KNN	46.4	86.9	21.6	0.11	0.56
RF	67.0	40.5	83.2	0.27	0.72

**Figure 1:**
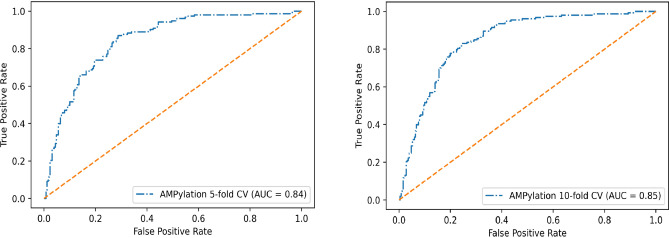
Receiver operating characteristic (ROC) curves for 5-fold CV and 10-fold CV DeepAmp model.

In Fig. 1, the receiver operating characteristic curves (ROC curves) clearly illustrate the capability of distinguishing the AMPylation and non-AMPylation sites of the DeepAmp model. To provide further information for the readers, the ROC curve for fivefold and tenfold cross validation (ROC curve for each fold) are also provided as supplementary materials (Figs. S1, S2). Also, as shown in Tables 1 and 2, in terms of the MCC score, the other ML models display mediocre classification quality, conversely, DeepAmp shows significant improvement in the classification quality. It demonstrate the effectiveness of DeepAmp over other classifiers in identification of positive and negative samples, consistently.

## Methods and materials

This section describes the proposed method and benchmark dataset presented in this study.

### Benchmark dataset

Kielkowski et al.^9^ has identified the AMPylation in intact cancer cells via LC-MS/MS as well as imaging methods. Using a pronucleotide probe they identified the protein AMPylation in living cells. They synthesized an N6-propargyl adenosine phosporamidate proneucleotide (pro-N6pA) and treated different cell lines such as HeLa, SH-SY5Y, etc. to identify the sites. The AMPylated proteins found here are engaged in a variety of metabolic pathways, including a widely conserved key regulator of glycolysis ATP-dependent 6-phosphofructokinase (PFKP), proteolysis (CTSA, CTSB), regulation of PTMs (PPME1), and UPR (HSPA5 and SQSTM1). They identified a total of 162 protein sequences to be involved in this distinct modification. We investigated these proteins through UniProt database and identified a total of 133 unique protein sequences which are used to build our dataset.

We then use CD-Hit to remove proteins with over 40% sequential similarities to discard redundancy in the dataset^31^. The resulting dataset contains 130 unique proteins with less than 40% sequential similarities. After that, for each AMPylation and non AMPylation site, a 31-residue peptide containing central AMPylation /non AMPylation site with 15 residues upstream and 15 residues downstream was extracted. We tried different length of peptide-containing which among them, using 31-residue peptides attained the best results. To build the peptides sequence for AMPylation sites at the two ends of the proteins with less than 15 neighboring amino acids on each side, we use equalized by padding with “X” residue. As a result, a total of 153 peptides with AMPylated sites and 28872 peptides with non-AMPylated sites were extracted from 130 protein sequences. From the 28872 non-AMPylated sites, we selected 250 sequences randomly to balance our dataset having almost 2:1 ratio of negative to positive samples. Thus our final dataset of 403 peptide sequences with 153 AMPylated peptides and 250 non- AMPylated peptides was created. This dataset is available at https://github.com/MehediAzim/DeepAmp.

### Feature encoding

**Figure 2:**
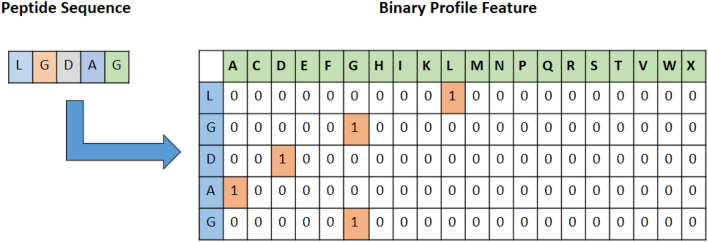
Binary profile feature generation of peptide sequences.

Feature encoding is an important step in building an effective machine learning model. Binary profile features (also known as one-hot-encoding) are straightforward, yet shown to be very effective for the prediction of different functionalities in the multi-omics dataset^32,33^. In this study, we generate Binary profiles for each peptide, by representing each amino acid as a vector of 20 dimensions in term of one hot encoding. For instance, Alanine is replaced by a 20 size one hot vector which is [1,0,0,0,0,0,0,0,0,0,0,0,0,0,0,0,0,0,0,0]. As a result, a sequence of length L was represented by a vector of dimensions L 20. Considering L= 31 (length of peptides), we extract 620 features for each peptide (31 20). This feature encoding process is depicted in Fig. 2. Considering that we use convolutional neural network to build our model, binary profile can potentially provide extensive information to train our model.

### Classification technique

Convolutional neural network (CNN) is widely used in computational biology for predicting different biological and chemical functionalities and entities from multi-omics datasets. It has shown tremendous success in the prediction of different PTMs, cancer cell types classification tasks, origins of replication prediction, and many more^34–36^. Like any other neural network, a CNN consists of an input layer, hidden layer, and an output layer. Extracting feature maps using convolution operation makes the CNN architecture different from the regular neural nets. Unlike hidden layers of regular neural net which basically constructed by a set of fully connected neurons, the hidden layers of CNN mainly consist of a convolutional layer, pooling layer, and fully connected layer^37^.

**Figure 3:**
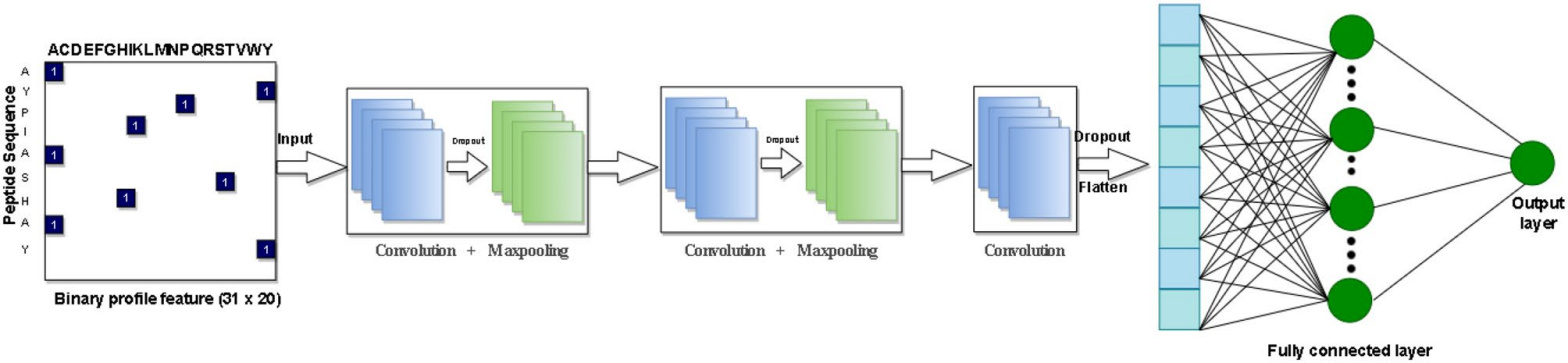
Model architecture of DeepAmp.

The CNN architecture we used is depicted in Fig. 3. Our CNN classifier consists of three Conv1D layers with the number of filters and kernel sizes of [24, 7], [16, 5] and [8, 3], respectively. We also use two Maxpooling1D layers as well as two Dense layers. The input is the L 20 matrix where L is the length of the protein sequence (31). We applied one-dimensional kernels to the input vectors. The output of our first 1-D convolutional layer which can also be thought of as a motif scanner is then passed to the max-pooling layer. Among the three convolutional layers we used, max-pooling was applied in the first two of them. The last convolutional layer output is directly passed to a fully connected layer and the prediction layer. Rectified Linear Unit (ReLU) was used as activation function for each intermediate layer as it is popularly used for its simplicity and effectiveness^38,39^. In each of the convolutional layers and the fully connected layer, we used dropout to avoid overfitting^40^.

Even though for computer vision problems deeper CNN models provide the best result^41^, for biological sequence data which are presented in term of matrix as input, different studies have shown that increasing the depth of the convolutional layer does not necessarily lead to improvement in prediction accuracy specially for the smaller datasets similar to ours^42^. Furthermore, it reduces the chance of overfitting and requires fewer instances for training^40,43^. In order to prevent overfitting, we develop a shallow CNN architecture. With only 7825 trainable parameters the model provides a balanced result. Additionally, to prevent the overfitting we use two regularization methods namely, dropout and L2 for each Conv1D.

### Evaluation methods

In order to measure the efficacy of DeepAmp, k-fold cross-validation is used here. In k-fold cross-validation, the dataset is split into k subsets. From this k subset, k-1 is used for training and the remaining fold is used for validation. This way the whole dataset gets used for training. Since the training size gets bigger, the classifiers tend to show better results. We used stratified k-fold cross validation which maintains a fixed ratio of negative and positive sites in the training and validation dataset^44^. In this study, we evaluate our model using k = 5 and 10 as two common values for this parameter.

## Conclusion

In this study, we presented a new dataset that can be used to evaluate computational methods specially machine learning based models to predict AMPylation PTM. On top of that, we proposed a new deep learning-based tool called DeepAmp for predicting AMPylation using CNN and binary profile feature vector. DeepAmp achieves an accuracy of 77.7% and sensitivity, specificity, MCC, and AUC score of 79.1%, 76.8%, 0.55, and 0.85 respectively for tenfold cross-validation. DeepAmp also significantly outperforms widely used machine learning models including Support Vector Machine, K-nearest Neighbor, and Random Forest for predicting AMPylation sites. Due to the limitation of the sample size available, prediction with high accuracy is strenuous. In the future refinement of our work, we aim to incorporate new AMPylation sites into the dataset and create a larger database for AMPylation PTM. Furthermore, we aim to ameliorate our predictor’s performance by using different feature sets and deeper CNN architectures. Our presented dataset and DeepAmp as an standalone predictor are publicly available at https://github.com/MehediAzim/DeepAmp.

## Supplementary Information

Supplementary Figures.

## References

[1] Jensen ON (2004). Modification-specific proteomics: Characterization of post-translational modifications by mass spectrometry. Curr. Opin. Chem. Biol..

[2] Kia-Ki H, Martinage A (1992). Post-translational chemical modification (s) of proteins. Int. J. Biochem..

[3] Lin J, Liang H, Yan J, Luo L (2017). The molecular mechanism and post-transcriptional regulation characteristic of *Tetragenococcus halophilus* acclimation to osmotic stress revealed by quantitative proteomics. J. Proteomics.

[4] Kristiansen K (2004). Molecular mechanisms of ligand binding, signaling, and regulation within the superfamily of g-protein-coupled receptors: Molecular modeling and mutagenesis approaches to receptor structure and function. Pharmacol. Therap..

[5] Chauhan, M., Tarique, M., Sourabh, S. & Tuteja, R. Overview of posttranslational modifications of biochemically characterized plasmodium falciparum helicases. In *Helicases from All Domains of Life*, 113–124 (Elsevier, 2019).

[6] Yarbrough ML (2009). Ampylation of rho gtpases by vibrio vops disrupts effector binding and downstream signaling. Science.

[7] Yarbrough ML, Orth K (2009). Ampylation is a new post-translational modification. Nat. Chem. Biol..

[8] Casey AK, Orth K (2018). Enzymes involved in ampylation and deampylation. Chem. Rev..

[9] Kielkowski P (2020). Ficd activity and ampylation remodelling modulate human neurogenesis. Nat. Commun..

[10] Mullard A (2009). Examining the fic domain. Nat. Rev. Microbiol..

[11] Itzen A, Blankenfeldt W, Goody RS (2011). Adenylylation: Renaissance of a forgotten post-translational modification. Trends Biochem. Sci..

[12] Anderson WB, Stadtman E (1970). Glutamine synthetase deadenylylation: A phosphorolytic reaction yielding adp as nucleotide product. Biochem. Biophys. Res. Commun..

[13] Rahman M (2012). Visual neurotransmission in drosophila requires expression of fic in glial capitate projections. Nat. Neurosci..

[14] Ham H (2014). Unfolded protein response-regulated drosophila fic (dfic) protein reversibly ampylates bip chaperone during endoplasmic reticulum homeostasis. J. Biol. Chem..

[15] Sieber SA, Cappello S, Kielkowski P (2020). From young to old: AMPylation hits the brain. Cell Chem. Biol..

[16] Choudhary C, Weinert BT, Nishida Y, Verdin E, Mann M (2014). The growing landscape of lysine acetylation links metabolism and cell signalling. Nat. Rev. Mol. Cell Biol..

[17] Nishida Y (2015). Sirt5 regulates both cytosolic and mitochondrial protein malonylation with glycolysis as a major target. Mol. Cell.

[18] Du Y (2015). Lysine malonylation is elevated in type 2 diabetic mouse models and enriched in metabolic associated proteins. Mol. Cell. Proteomics.

[19] Xie Z (2012). Lysine succinylation and lysine malonylation in histones. Mol. Cell. Proteomics.

[20] Olsen CA (2012). Expansion of the lysine acylation landscape. Angew. Chem. Int. Ed..

[21] Azim SM, Haque MR, Shatabda S (2021). Oric-ens: A sequence-based ensemble classifier for predicting origin of replication in *S. cerevisiae*. Comput. Biol. Chem..

[22] Peng C (2011). The first identification of lysine malonylation substrates and its regulatory enzyme. Mol. Cell. Proteomics.

[23] Hirschey MD, Zhao Y (2015). Metabolic regulation by lysine malonylation, succinylation, and glutarylation. Mol. Cell. Proteomics.

[24] Reddy HM (2019). Glystruct: Glycation prediction using structural properties of amino acid residues. BMC Bioinform..

[25] Dipta SR (2020). Semal: Accurate protein malonylation site predictor using structural and evolutionary information. Comput. Biol. Med..

[26] Chandra A (2018). Phoglystruct: Prediction of phosphoglycerylated lysine residues using structural properties of amino acids. Sci. Rep..

[27] Uddin MR (2018). Evostruct-sub: An accurate gram-positive protein subcellular localization predictor using evolutionary and structural features. J. Theor. Biol..

[28] Taherzadeh G, Dehzangi A, Golchin M, Zhou Y, Campbell MP (2019). Sprint-gly: Predicting n-and o-linked glycosylation sites of human and mouse proteins by using sequence and predicted structural properties. Bioinformatics.

[29] Khater S, Mohanty D (2015). In silico identification of ampylating enzymes and study of their divergent evolution. Sci. Rep..

[30] Rosner B (2015). Fundamentals of Biostatistics.

[31] Huang Y, Niu B, Gao Y, Fu L, Li W (2010). Cd-hit suite: A web server for clustering and comparing biological sequences. Bioinformatics.

[32] Yi H-C (2019). Acp-dl: A deep learning long short-term memory model to predict anticancer peptides using high-efficiency feature representation. Mol. Therapy-Nucleic Acids.

[33] Xiao X, Shao S, Ding Y, Huang Z, Chou K-C (2006). Using cellular automata images and pseudo amino acid composition to predict protein subcellular location. Amino Acids.

[34] Le NQK, Ho Q-T, Nguyen T-T-D, Ou Y-Y (2021). A transformer architecture based on bert and 2d convolutional neural network to identify dna enhancers from sequence information. Brief. Bioinform..

[35] Bhinder B, Gilvary C, Madhukar NS, Elemento O (2021). Artificial intelligence in cancer research and precision medicine. Cancer Discov..

[36] Oberti M, Vaisman II (2020). cnnalpha: Protein disordered regions prediction by reduced amino acid alphabets and convolutional neural networks. Proteins Struct. Funct. Bioinform..

[37] Krizhevsky A, Sutskever I, Hinton GE (2012). Imagenet classification with deep convolutional neural networks. Adv. Neural. Inf. Process. Syst..

[38] Yarotsky D (2017). Error bounds for approximations with deep relu networks. Neural Netw..

[39] Li, Y. & Yuan, Y. Convergence analysis of two-layer neural networks with relu activation. Preprint at http://arxiv.org/abs/1705.09886 (2017).

[40] Srivastava N, Hinton G, Krizhevsky A, Sutskever I, Salakhutdinov R (2014). Dropout: A simple way to prevent neural networks from overfitting. J. Mach. Learn. Res..

[41] LeCun Y, Bengio Y, Hinton G (2015). Deep learning. Nature.

[42] Min S, Lee B, Yoon S (2017). Deep learning in bioinformatics. Brief. Bioinform..

[43] Ioffe, S. & Szegedy, C. Batch normalization: Accelerating deep network training by reducing internal covariate shift. In *International Conference on Machine Learning*, 448–456 (PMLR, 2015).

[44] He H, Ma Y (2013). Imbalanced Learning: Foundations, Algorithms, and Applications.

