# Supplementary material for: A convolutional neural network based tool for predicting protein AMPylation sites from binary profile representation
Source: Sci Rep. 2022 Jul 6;12:11451. doi: 10.1038/s41598-022-15403-3 (PMC9259580; doi:10.1038/s41598-022-15403-3)

Supplementary Materials

Figure S1: The ROC curve for 5-fold validation (ROC curve for each fold)

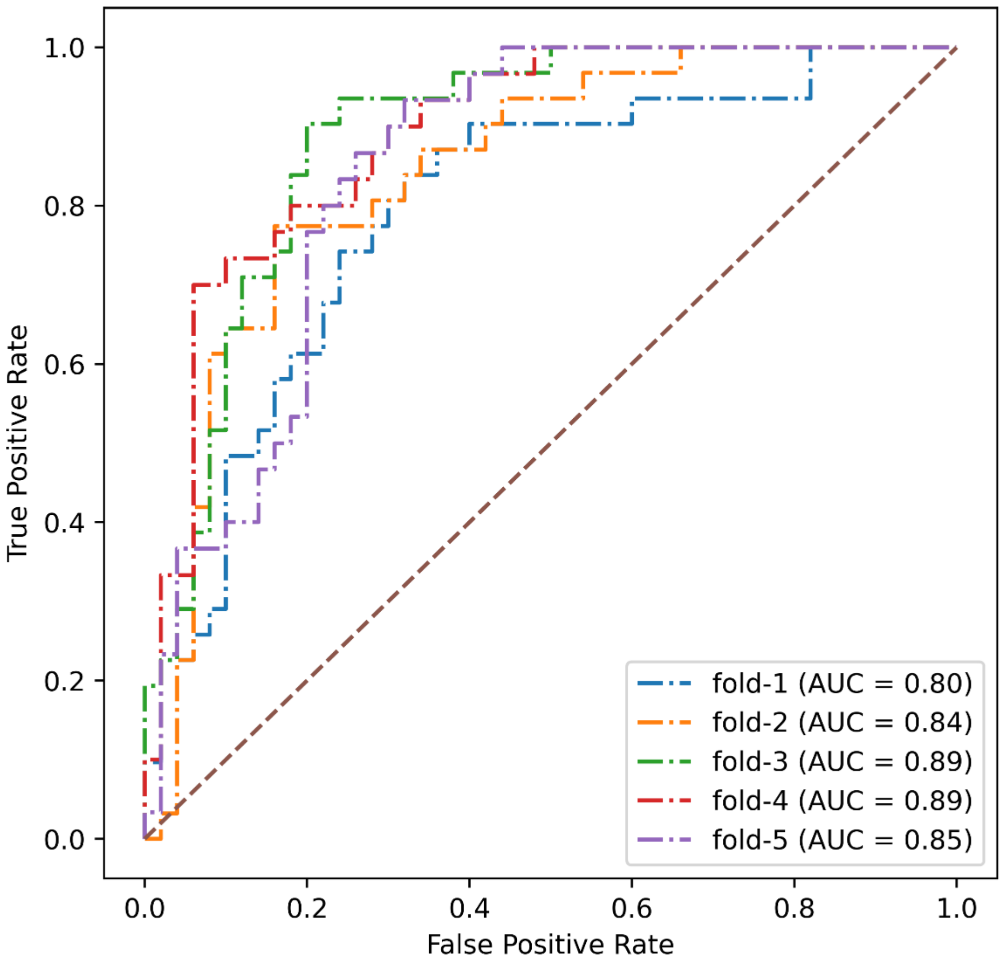

Figure S2: The ROC curve for 10-fold cross validation (ROC curve for each fold)

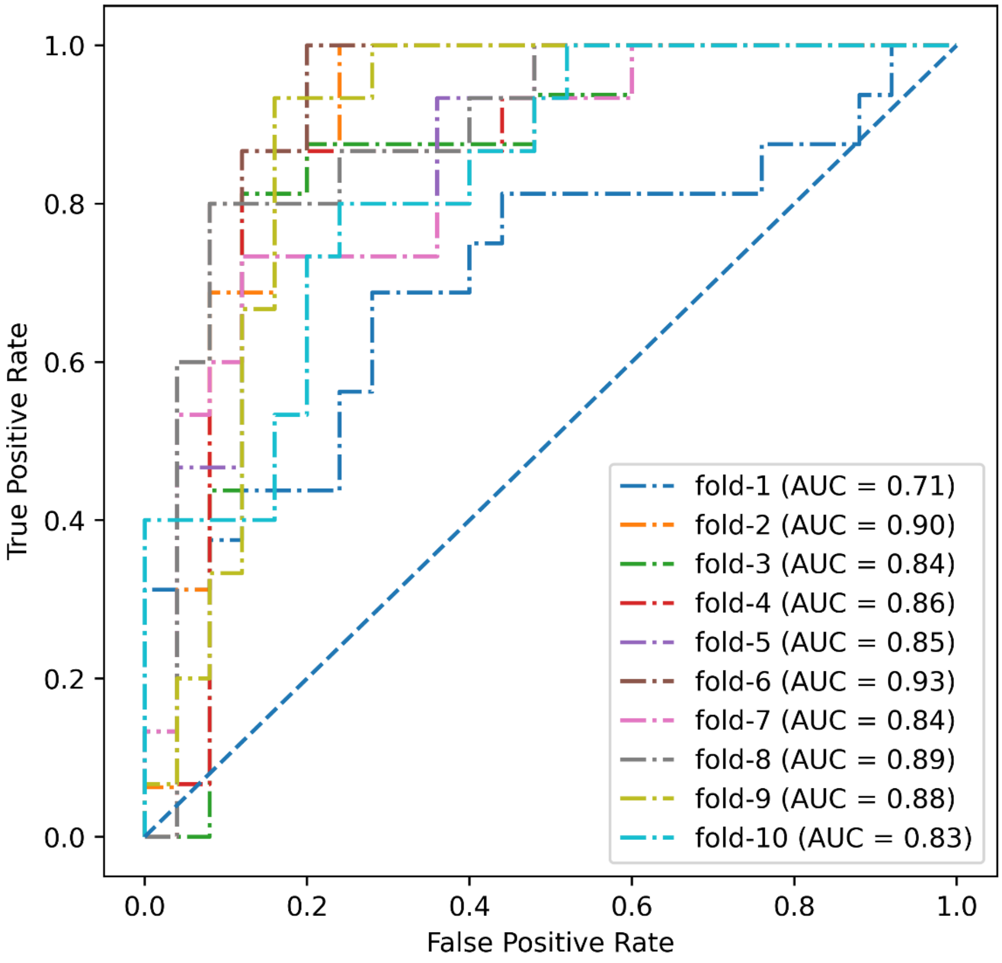

Supplement: Supplementary file 1 — Supplementary Figures. [file 41598_2022_15403_MOESM1_ESM.pdf]
